# Author Correction: Promoter interactome of human embryonic stem cell-derived cardiomyocytes connects GWAS regions to cardiac gene networks

**DOI:** 10.1038/s41467-018-07399-0

**Published:** 2018-11-12

**Authors:** Mun-Kit Choy, Biola M. Javierre, Simon G. Williams, Stephanie L. Baross, Yingjuan Liu, Steven W. Wingett, Artur Akbarov, Chris Wallace, Paula Freire-Pritchett, Peter J. Rugg-Gunn, Mikhail Spivakov, Peter Fraser, Bernard D. Keavney

**Affiliations:** 1 Division of Cardiovascular Sciences, The University of Manchester, Manchester, M13 9PT UK; 2 Nuclear Dynamics Programme, The Babraham Institute, Cambridge, CB22 3AT UK; 3 Josep Carreras Leukaemia Research Institute, Campus ICO-Germans Trias I Pujol, Badalona, 08916 Barcelona, Spain; 4 MRC Biostatistics Unit, University of Cambridge, Cambridge, CB2 0SR UK; 5 Department of Medicine, University of Cambridge, Cambridge, CB2 0QQ UK; 6 Division of Cell Biology, Medical Research Council Laboratory of Molecular Biology, Cambridge, CB2 0QH UK; 7 Epigenetics Programme, The Babraham Institute, Cambridge, CB22 3AT UK; 8 Department of Biological Science, Florida State University, Tallahassee, 32306 FL USA

Correction to: *Nature Communications* 10.1038/s41467-018-04931-0; published online 28 June 2018

In the original version of the Article, the gene symbol for tissue factor pathway inhibitor was inadvertently given as ‘*TFP1*’ instead of ‘*TFPI*’. This has now been corrected in both the PDF and HTML versions of the Article.

