# Supplementary material for: Promoter interactome of human embryonic stem cell-derived cardiomyocytes connects GWAS regions to cardiac gene networks
Source: Nat Commun. 2018 Jun 28;9:2526. doi: 10.1038/s41467-018-04931-0 (PMC6023870; doi:10.1038/s41467-018-04931-0)

**Promoter interactome of human embryonic stem cell-derived cardiomyocytes connects GWAS regions to cardiac gene networks**

**Choy *et al*.**

Supplementary Table 1. List of genes involved in top 20% hESC-CM promoter interactions for read counts and found significantly enriched in canonical pathways of cardiovascular development or functions (Benjamini-Hochberg (B-H) *p* values < 0.005) using Ingenuity Pathway Analysis (Qiagen).

| Ingenuity canonical pathway | -log(B-H *p* value) | Ratio | Gene |
| --- | --- | --- | --- |
| Cardiac hypertrophy signalling | 5.68 | 0.292 | *MAPK1, ADCY4, PIK3R1, DIRAS3, TGFBR2, GNB1, ROCK2, RHOG, CACNA1E, RHOB, ADCY5, MRAS, IRS2, GSK3B, PLCL1, FRS2, ADRA1B, PIK3C2B, IL6R, CREBBP, FGFR2, PPP3CC, MEF2B, ADRA1D, ADCY9, ADRA2A, RND3, GAB1, PTPN11, IRS1, PRKACG, PRKACA, TGFB3, GNB2, MAP2K3, RPS6KA1, PIK3CA, MAP3K5, EIF4E, PLCH1, PRKAG1, EP300, HAND2, JUN, IGF1, RHOT1, RHOD, IGF1R, TGFB2, PRKAR1B, ADCY8, MAP2K1, MYL12B, ADCY2, GNAS, BORCS8-MEF2B, MAPKAPK3, RHOC, GRB2, MYLPF, MAP3K1, MEF2A, PIK3C2G, GNAQ, GNAI1, MAPK9, GNAI2, CALM1* (includes others)*, PLCB4* |
| Role of NFAT in cardiac hypertrophy | 5.01 | 0.295 | *LIF, MAPK1, ADCY4, PIK3R1, CABIN1, TGFBR2, GNB1, ADCY5, MRAS, IRS2, GSK3B, PLCL1, FRS2, PIK3C2B, FGFR2, PPP3CC, MEF2B, ADCY9, GAB1, PTPN11, IRS1, PRKACG, PRKACA, GNB2, TGFB3, MAP2K3, CAMK2G, PIK3CA, PLCH1, PRKAG1, PRKCZ, EP300, IGF1, CAMK1G, IGF1R, TGFB2, PRKAR1B, PRKCE, ADCY8, MAP2K1, PRKCA, HDAC9, ADCY2, GNAS, BORCS8-MEF2B, GRB2, MAP3K1, MEF2A, GNAQ, GNAI1, PIK3C2G, MAPK9, GNAI2, RCAN1, CALM1* (includes others)*, PLCB4, RCAN2* |
| Factors promoting cardiogenesis in vertebrates | 3.96 | 0.337 | *TBX5, BMP15, TCF4, BMP4, FZD3, BMP3, TCF7L1, BMPR1B, FZD1, TCF3, BMP5, PRKCZ, APC, TGFBR2, FZD8, FZD4, BMPR1A, TGFB2, TGFB3, PRKCE, LEF1, FZD5, GSK3B, DKK1, CTNNB1, BMP6, FZD2, CDK2, TCF7L2, LRP1, PRKCA* |
| Cardiac β-adrenergic signalling | 3.37 | 0.286 | *AKAP12, NAPEPLD, AKAP8, PPP1R1A, PPP1R3C, PDE3A, PDE12, ADCY4, PPP1CB, PPP2R3B, PRKAG1, GNB1, CACNA1E, ADCY5, PPM1J, PPM1L, MRAS, PRKAR1B, TDP2, PPP2R5C, PDE4D, PKIA, ADCY8, PLN, PPP1R14C, PDE2A, ADCY2, GNAS, PDE10A, PPP2R5D, RYR2, ATP2A3, PPP1R11, ADCY9, TULP2, PRKACG, PRKACA, GNB2, PDE5A, PKIG* |
| Nitric oxide signalling in the cardiovascular system | 3.03 | 0.292 | *PIK3CA, MAPK1, PIK3R1, PRKAG1, PRKCZ, VEGFA, CACNA1E, PROK1, PRKAR1B, PRKCE, IRS2, MAP2K1, FRS2, PRKCA, PLN, PIK3C2B, PDE2A, GUCY1A3, GRB2, RYR2, PIK3C2G, ATP2A3, FGFR2, VEGFC, CALM1* (includes others)*, PTPN11, GAB1, IRS1, PRKACG, PRKACA, GUCY2F, GUCY1A2, PDE5A* |

Supplementary Fig. 1. Venn diagram showing the overlaps of promoter interactions in three biological replicates of hESC-CMs (a, b and c). The areas are proportional to the percentages.


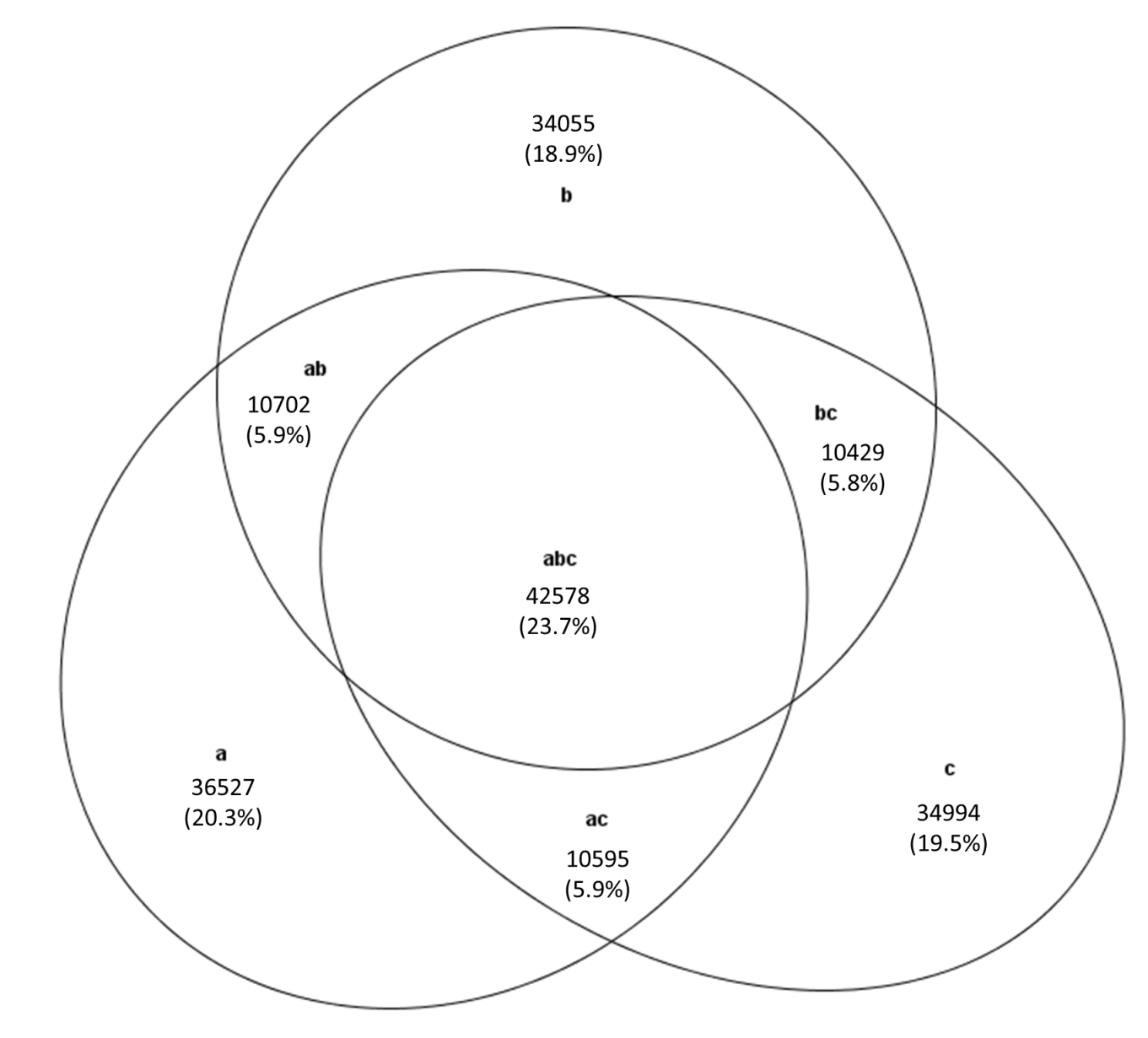


Supplementary Fig. 2. Uncropped images of the a) mm172 (gDNA), b) *Actb* (gDNA), c) *Ednra* (cDNA) and d) *Actb* (cDNA) PCR gels in Fig. 2d. The size marker is HyperLadder 1kb (Bioline).


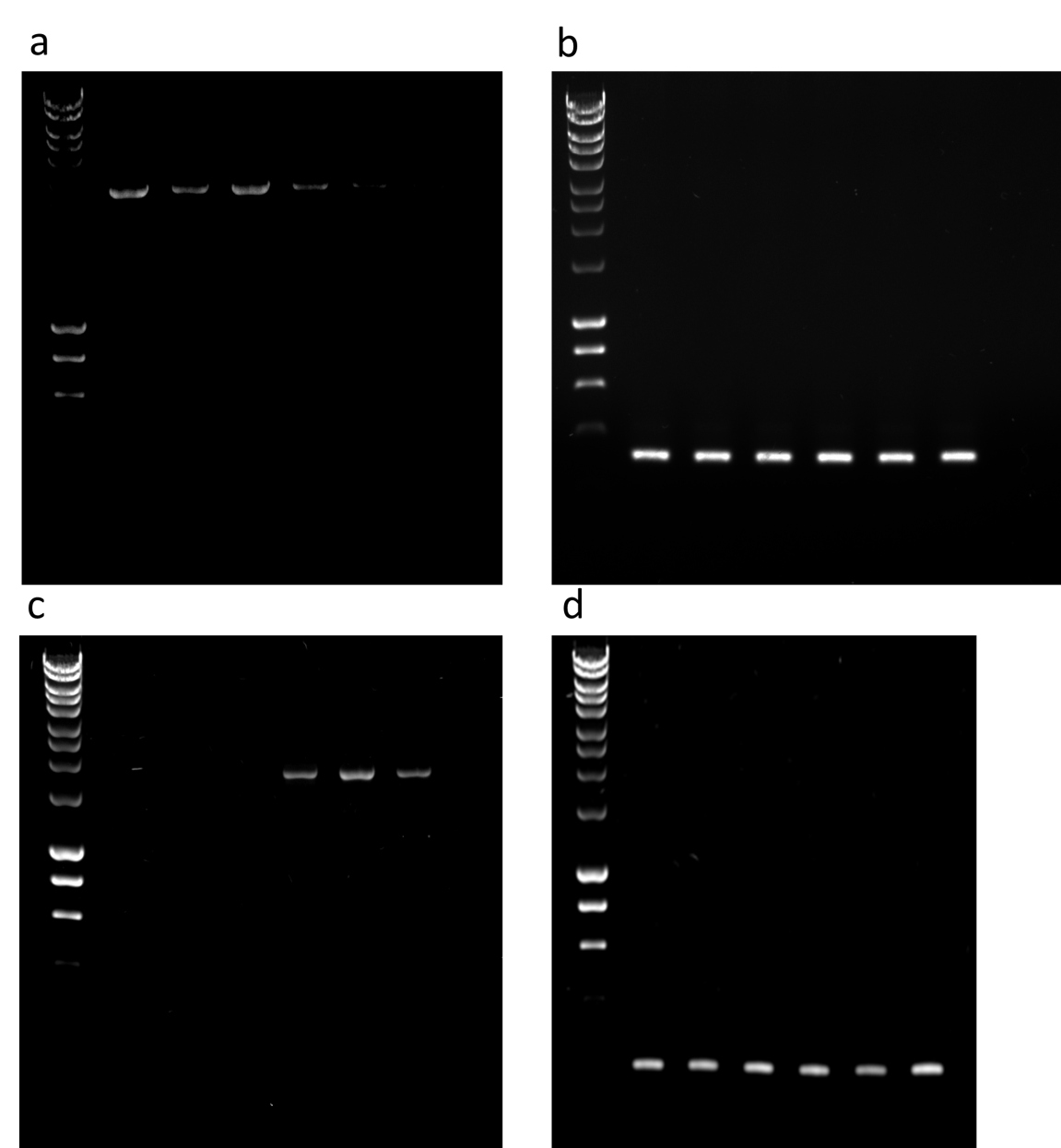


Supplementary Fig. 3. Uncropped images of the a) EDNRA and b) ACTB immunoblots in Fig. 2d. The size marker is Precision Plus Protein Dual Colour Standards (Bio-Rad).

a b


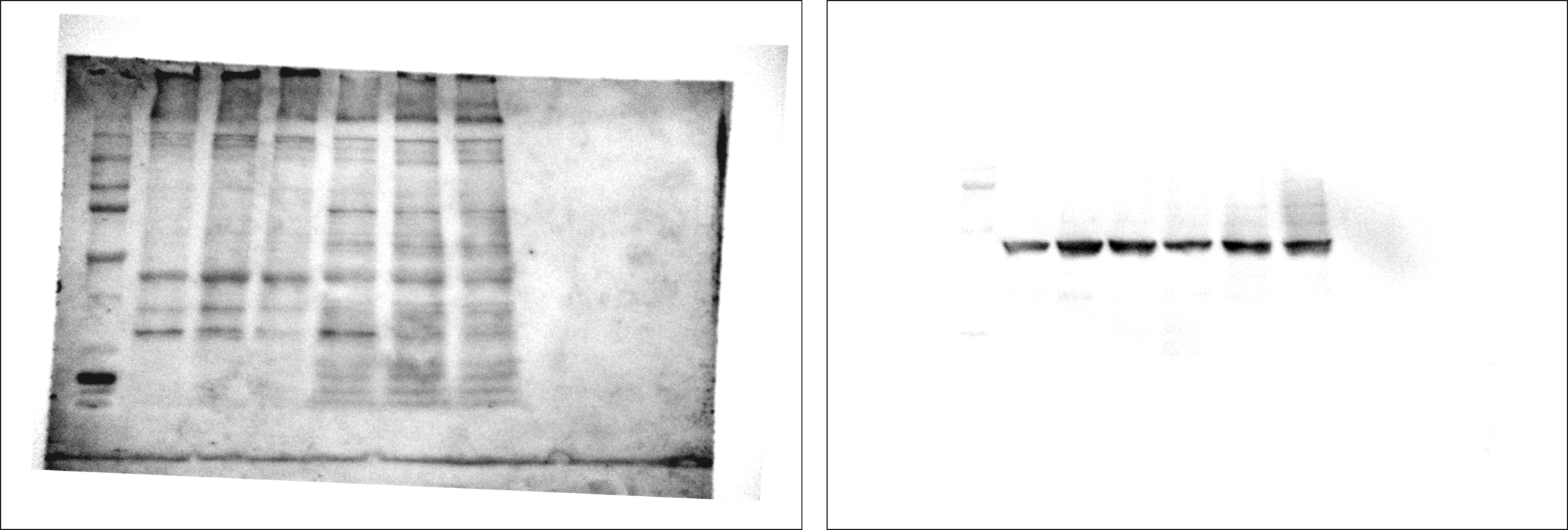

Supplement: Supplementary file 1 — Supplementary Information [file 41467_2018_4931_MOESM1_ESM.docx]
