# Supplementary material for: Promoter interactome of human embryonic stem cell-derived cardiomyocytes connects GWAS regions to cardiac gene networks
Source: Nat Commun. 2018 Jun 28;9:2526. doi: 10.1038/s41467-018-04931-0 (PMC6023870; doi:10.1038/s41467-018-04931-0)
Supplement: Supplementary file 3 — Description of Additional Supplementary Files [file 41467_2018_4931_MOESM3_ESM.pdf]

Description of Additional Supplementary Files

File Name: Supplementary Data 1
Description: List of 179880 significant hESC-CM promoter-genome interactions with average read counts from three biological replicates.

File Name: Supplementary Data 2
Description: List of cardiac VISTA enhancers (with their bracketing genes) that intersect with cPIRs (with their target genes).

File Name: Supplementary Data 3
Description: List of connected subnetworks constructed from top 20% of hESCCM promoter interactions for read counts.

File Name: Supplementary Data 4
Description: List of significant ($p < 1x10-5$) SNPs from the GWAS of cardiac conduction and rhythm disorders located within cPIRs and the genes they interact with.
